# A genetic dissection of the LlaJI restriction cassette reveals insights on a novel bacteriophage resistance system

**DOI:** 10.1186/1471-2180-6-40

**Published:** 2006-04-28

**Authors:** Jonathan O'Driscoll, Daniel F Heiter, Geoffrey G Wilson, Gerald F Fitzgerald, Richard Roberts, Douwe van Sinderen

**Affiliations:** 1 Department of Microbiology, University College Cork, Western Road, Cork, Ireland; 2 Alimentary Pharmabiotic Centre, University College Cork, Western Road, Cork, Ireland; 3 Biotransfer Unit, University College Cork, Western Road, Cork, Ireland; 4 New England Biolabs, 240 County Road, Ipswich, MA 01938-2723, USA

## Abstract

**Background:**

Restriction/modification systems provide the dual function of protecting host DNA against restriction by methylation of appropriate bases within their recognition sequences, and restriction of foreign invading un-methylated DNA, such as promiscuous plasmids or infecting bacteriphage. The plasmid-encoded LlaJI restriction/modification system from *Lactococcus lactis *recognizes an asymmetric, complementary DNA sequence, consisting of 5'GACGC'3 in one strand and 5'GCGTC'3 in the other and provides a prodigious barrier to bacteriophage infection. LlaJI is comprised of four similarly oriented genes, encoding two 5mC-MTases (M1.LlaJI and M2.LlaJI) and two subunits responsible for restriction activity (R1.LlaJI and R2.LlaJI). Here we employ a detailed genetic analysis of the LlaJI restriction determinants in an attempt to characterize mechanistic features of this unusual hetero-oligomeric endonuclease.

**Results:**

Detailed bioinformatics analysis confirmed the presence of a conserved GTP binding and hydrolysis domain within the C-terminal half of the R1.LlaJI amino acid sequence whilst the N-terminal half appeared to be entirely unique. This domain architecture was homologous with that of the "B" subunit of the GTP-dependent, methyl-specific McrBC endonuclease from *E.coli *K-12. R1.LlaJI did not appear to contain a catalytic centre, whereas this conserved motif; PD....D/EXK, was clearly identified within the amino acid sequence for R2.LlaJI. Both R1.LlaJI and R2.LlaJI were found to be absolutely required for detectable LlaJI activity *in vivo*. The LlaJI restriction subunits were purified and examined *in vitro*, which allowed the assignment of R1.LlaJI as the sole specificity determining subunit, whilst R2.LlaJI is believed to mediate DNA cleavage.

**Conclusion:**

The hetero-subunit structure of LlaJI, wherein one subunit mediates DNA binding whilst the other subunit is predicted to catalyze strand hydrolysis distinguishes LlaJI from previously characterized restriction-modification systems. Furthermore, this distinction is accentuated by the fact that whilst LlaJI behaves as a conventional Type IIA system *in vivo*, in that it restricts un-methylated DNA, it resembles the Type IV McrBC endonuclease, an enzyme specific for methylated DNA. A number of similar restriction determinants were identified in the database and it is likely LlaJI together with these homologous systems, comprise a new subtype of the Type II class incorporating features of Type II and Type IV systems.

## Background

The diverse structural and functional activities described for restriction endonucleases (REases) is reflected by their ubiquitous abundance among prokaryotic organisms [7]. These enzymes are typically components of restriction/modification (R/M) systems where they primarily serve as defensive instruments against bacteriophage infection and other invading "foreign" DNA.

Four distinct groups of REases are known, with the "Type II" group being the most richly represented [8]. Members of this group, of which there are eleven subtypes, generally recognize relatively short DNA sequences and specifically cut within or reasonably close to such sequences in a Mg^+2 ^dependent manner.

Although generally lacking significant amino acid sequence homology, structural analysis of many of these REases has revealed in some instances a conserved core, consisting of a four/five-stranded mixed β-sheet flanked by α-helices, reviewed in [9]. This core spatially localizes two carboxylates (present in the amino acids D and D/E) which are believed to be involved in divalent metal ion binding, and a lysine residue, possibly involved in positioning and/or activation of an attacking nucleophile [10]. Together it is believed that these residues comprise the REase catalytic site, the PD...D/EXK motif, responsible for phosphodiester bond hydrolysis in the target DNA molecule.

For double-stranded scission this hydrolysis event must occur on both strands of the target DNA thereby requiring two catalytic sites [11], although single-site exceptions do exist [12,13]. Consequently, the active forms of many Type II REases are homodimers (or even tetramers), with each identical subunit providing a single catalytic site for individual strand hydrolysis [9,14]. Of course given the multifarious nature of the Type II group, it is not surprising that unusual hetero-multimeric REases, comprised of unique subunits, consisting of the associated products of different genes, have been described [1,3,15-18].

One such characterized enzyme, BbvCI, has been shown to be heterodimeric, with each individual subunit independently responsible for cleavage of separate strands of its asymmetric recognition sequence [15,19]. Similarly, the Bpu10I REase requires two different subunits for its activity however, in this instance, a direct interaction between the subunits has yet to be completely established [17].

Previously we have described the cloning and transcriptional regulation of LlaJI R/M system [1,20]. The LlaJI gene cluster, encoded by the lactococcal plasmid pNP40, consists of two 5mC MTase-encoding genes followed by two REase-encoding genes which mediate a prodigious barrier to bacteriophage infection [1]. The specificity of the system was determined to be asymmetric but complementary, comprised of 5'GACGC'3 in one strand and 5'GCGTC'3 in the other [20]. Perhaps the most striking feature of this R/M system is its homology to the methyl-specific REase McrBC. This highly unusual enzyme, which lacks an associated MTase, has been characterized extensively [10,21-31]. Its orphan nature, unusual specificity and cofactor requirements define McrBC, together with its identified homologues, as a distinct REase group [8].

The *mcrBC *locus encodes two major products, McrB (53 kDa) and McrC (39 kDa), which mediate the restriction phenotype [26]. McrBC recognizes two methylated/hemimethylated R^m^C sites (separated by 40 bp to up to 3000 bp) and cleaves at random positions between the two [24,25,32]. The optimal stoichiometry for cleavage *in vitro *has been suggested as 3–5:1, McrB:McrC (although a 1:1 ratio has been reported by another group [10]), with the cleavage reaction strictly dependent upon GTP and Mg^+2 ^[25,33].

The McrB subunit harbours the specific DNA binding activity (which resides within its N-terminus), in addition to GTP binding and hydrolysis activities [22,30,34]. The McrC subunit contains the catalytic centre for cleavage [10]. Following binding to its methylated target site, McrBC facilitates a GTP-dependent translocation of the DNA, akin to the mechanisms of other NTP-dependent REases of the Type I and III groups, with cleavage ensuing upon stalling of the translocating complex [23,35]. Furthermore, it has been shown that McrB forms high molecular weight complexes – heptameric rings, in the presence of GTP (although independent of GTP hydrolysis). These complexes can further multimerise to tetradecamers, structures stabilized by the binding of McrC – which constitutes the "active" form of the endonuclease [31].

In this report, we execute a detailed genetic and phenotypic assessment of the McrBC-like LlaJI endonuclease which serves to portray the complexity of this unique enzyme.

## Results

### Sequence analysis of LlaJI

**Figure 1:**
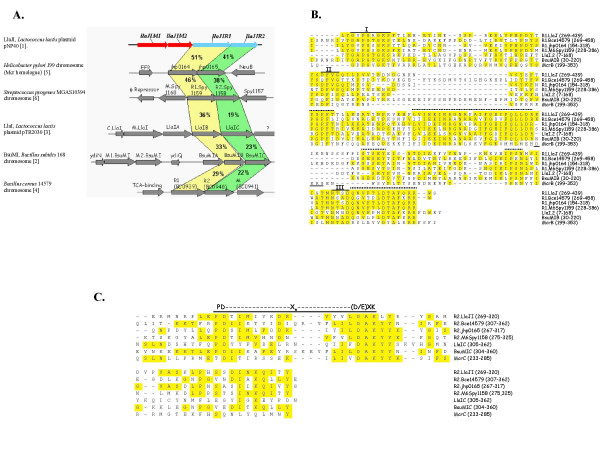
A: LlaJI and its identified homologous restriction modification systems. Genes sharing protein sequence homology to LlaJI components are linked by shading (with the percentages representative of the respective identity to LlaJI). B: Alignment of R1.LlaJI with is identified homologues. The aligned regions correspond to a section containing the three conserved motif's (indicated above each on the alignment) which comprise the predicted GTP binding and hydrolysis domain. Motif II for McrB is underlined. Additional conserved modules in this region are indicated by dashed lines above each. C: Alignment of the section of R2.LlaJI and its identified homologues predicted to contain the catalytic centre. The conserved PD...Xn....(D/E)XK motif is indicated.

R1.LlaJI is predicted to be a 67.2 kDa protein which exhibits sequence homology to a number of partially characterized and putative REases in the database (see Figure 1A and 1B). These homologies primarily correspond to a region proposed to represent a domain responsible for GTP binding and hydrolysis (Fig 1B). This domain consists of three signature motifs, namely (1) the phosphate binding loop (GxxxxGK(S/T)), (2) the switch II region (DXXG), and (3) the guanine recognition loop (NKxD) [36].

This domain has been investigated biochemically in the case of the R1.LlaJI homologue, the McrB subunit of the McrBC endonuclease – and has revealed interesting variations from the archetypal GTPase family (see discussion) [29].

Additional conserved modules (of identity) can be discerned within R1.LlaJI and its homologues; however, at this point no functional significance can be assigned to any of these regions (Fig 1B). It was suggested for McrB, that some of these conserved modules, at the time identified using completely different protein sets, may contribute in some manner to the diverged functionality of the GTP-binding domain [29]. Hence, it is likely that, as for McrB, R1.LlaJI represents yet another member of a distinct subclass of the classical GTPase family.

For most of the identified R1.LlaJI homologues, significant alignment was only achieved with portions of the sequences corresponding to the C-terminal half of each, with the N-terminal portions apparently representing non-conserved sequence. The two exceptions to this, namely LlaIB and BsuMIB (Fig 1A and 1B), which are smaller (337 and 343 aa's, respectively) than R1.LlaJI (585 aa), aligned completely along the full length of their respective sequences. Interestingly, both appear to be encoded by "restriction cassettes" comprised of three ORF's as opposed to the two observed for the remaining homologues (Fig 1A).

Database searches with R2.LlaJI (48.8 kDa) revealed a set of similar proteins that are encoded by genes located adjacent to those that had been identified as encoding homologues of R1.LlaJI (see Fig 1A and 1C). 1A multiple alignment of R2.LlaJI with its homologues allowed the identification of a putative catalytic centre for DNA cleavage; PD...D/ExK. This assignment is supported by inclusion of the McrC subunit of McrBC in the alignment, whose catalytic centre has essentially been confirmed [10]. As for R1.LlaJI above, R2.LlaJI shares significant, but scattered modules of identity with other proteins, but again the functional significance of this similarity remains purely speculative.

These data cumulatively suggest that LlaJI, and indeed other restriction systems exhibit structural and therefore likely mechanistic similarities with that of the McrBC methyl-specific endonuclease (see discussion).

### Phenotypic and genetic analysis of LlaJI

Previously, we have described the bacteriophage resistance phenotype conferred by the LlaJI operon [1]. To fully explore the contribution of the individual LlaJI restriction "subunits" to this phage resistance activity, we adopted a genetic subcloning approach. To this end, the *llaJIR1 *and *llaJIR2 *genes were amplified individually, and in tandem, and placed under the control of the strong nisin-inducible promoter of plasmid pNZ8020 to facilitate their over-expression.

The resulting constructs, pNZ-R1, pNZ-R2, and pNZ-R1R2 were subsequently introduced into the expression host NZ9000, containing the plasmid pJO-M1M2 (which encodes both LlaJI MTases), and analyzed for their ability to confer phage resistance. As can be seen from Table 2, it was apparent that both R1.LlaJI and R2.LlaJI were required for the complete LlaJI phenotype (an observation we had previously alluded to [1]). Furthermore, at 30°C the degree of phage resistance exhibited by the host over-expressing both LlaJI subunits (on plasmid pNZ-R1R2) was 100-fold greater than that exhibited by the "wild type" LlaJI operon present on the low copy number plasmid pJO-J. Notably, the significant decrease in EOP conferred by the host containing pJO-J, which accompanied growth at 19°C, the previously established optimum for LlaJI-mediated resistance, was not preserved in the pNZ-R1R2 containing host. In fact, at 30°C the degree of LlaJI-mediated resistance conferred by pNZ-R1R2 was comparable to that obtained at 19°C with the pJO-J containing host, which would suggest that the upper threshold for LlaJI activity had been reached.

The fact that this optimal level of resistance may be achieved at 30°C by mere over-expression of R1.LlaJI and R2.LlaJI suggests that the temperature sensitivity of the LlaJI system previously reported, may be attributable to the intracellular concentration of active LlaJI at the respective temperatures (modulated by the LlaJI natural transcriptional signals), rather than to the temperature "activity" spectrum of assembled functional LlaJI, as had previously been speculated [1].

As stated, the results obtained with the hosts expressing R1.LlaJI and R2.LlaJI individually (on plasmids pNZ-R1, and pNZ-R2, respectively) indicated that neither subunit alone was capable of restricting the growth of phage. However, despite the lack of significant decrease in EOP at either sampling temperature used, a delicate but visibly obvious variable plaque phenotype was observed for the host expressing R1.LlaJI alone (see Table 2). Perhaps most intriguing was the complete absence of any detectable endonuclease activity in crude extracts of lactococcal strains containing either pJO-J or the pNZ-R1R2 over-expression plasmid (which as stated was always accompanied with plasmid pJO-M1M2).

### Purification of the individual LlaJI subunits

As it was established that both LlaJI subunits were required for "complete" activity, it was assumed that expression of the individual subunits separately for purification purposes could be achieved in the absence of the LlaJI MTases. To this end, both *llaJIR1 *and *llaJIR2 *were cloned in plasmid pQE60 to produce derivatives pQE-R1 and pQE-R2, respectively which were subsequently established in *E.coli *(see materials and methods). Over-expression with pQE-R1 allowed purification of R1.LlaJI as a C-terminal 6xhis tagged derivative. Mass spectrometry and N-terminal sequencing confirmed the predicted molecular mass (68.139 kDa inclusive of the mass of the 6x His tag) and identity of purified R1.LlaJI.

Purification of R2.LlaJI by this approach failed, possibly due to instability of the resulting recombinant protein. To overcome this presumed instability, *llaJIR2 *was inserted downstream of the *malE *gene of plasmid pMal-c2x under the control of the Ptac promoter (see materials and methods) which, upon induction resulted in the expression of an R2-MBP fusion of the predicted molecular mass (~90.8 kDa). This fusion was purified by affinity chromatography using amylose resin and the R2.LlaJI subunit was subsequently released from the R2-MBP preparation by cleavage with factor Xa protease. Rebinding of MBP to amylose resin resulted in purification of R2.LlaJI to ~60 % homogeneity.

### Assignment of the specificity determining LlaJI subunit

Given the domain architecture shared between R1.LlaJI and McrB, and the established DNA binding activity of the latter, we investigated the possibility of R1.LlaJI constituting the DNA recognition element of LlaJI.

**Figure 2:**
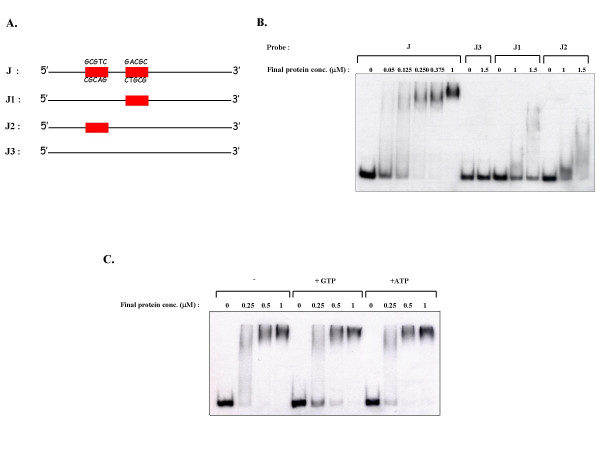
A: Schematic representation of the 230 bp DNA probes used for mobility shift assays. The positions of the intact LlaJI recognition sequences are indicated. B: Mobility shift assay with specific and non-specific probes (as indicated) which were incubated with increasing concentrations of purified R1.LlaJI. Final concentrations of protein are specified above each lane. C: Mobility shift assay with probe J which was incubated with increasing concentrations of purified R1.LlaJI (as indicated above each lane). The presence (+) or absence (-) of 5 mM GTP/ATP is as specified.

Mobility shift assays were therefore performed with R1.LlaJI using probes containing both single and multiple LlaJI sites, in addition to a non-specific probe (see materials and methods, and Figure 2A). As can be seen from Figure 2B, an obvious and specific DNA binding activity was evident using probes J1 and J2, each containing a single LlaJI recognition site. For both probes, binding did not result in a defined shifted band (with the R1.LlaJI concentrations used), but rather appeared as a "strewn" binding pattern, suggestive of a poorly defined protein:DNA stoichiometry of the retarded complex. At high R1.LlaJI concentrations, binding to probe J (which contained two LlaJI sites) resulted in the formation of more apparent yet still "diffuse" and extremely slow migrating complexes (Fig. 2B), reminiscent of the high molecular weight complexes formed by McrB with its specific probe [34]. Incubation of probe J3 (which is devoid of LlaJI sites) with excess R1.LlaJI did not reveal any binding activity (Fig 2B), and this finding, combined with competition experiments with unlabelled "specific" probes confirmed the specificity of the observed interactions (data not shown). Inclusion of Mg^+2 ^in the binding buffer had no discernable effect on R1.LlaJI specific binding, nor did its addition post protein:DNA complex formation. Interestingly however, and in contrast to McrB, inclusion of GTP did not stimulate the R1.LlaJI DNA interaction (Fig 2C). In fact it appeared that GTP, and to a lesser extent ATP, had a very modest inhibitory effect on the observed binding activity.

A similar analysis using partially purified R2.LlaJI did not reveal any DNA binding to the LlaJI site containing probes. In addition, R2.LlaJI did not appear to have any discernable effect on the R1.LlaJI binding activity.

### Investigation of the biological activity of the purified LlaJI subunits

As mentioned above, it was not possible to detect specific endonuclease activity in crude cell extracts of lactococcal strains (over)expressing the LlaJI system (as measured by its phage resistance phenotype). Subsequent attempts to reconstitute active LlaJI by combination of the partially purified subunits in various ratios, and with an assortment of cofactors (see materials and methods) failed to reveal any specific nuclease activity. Furthermore, neither subunit was found to exhibit a DNA nicking activity. Possible explanations for these observations are discussed below.

The presence of the putative GTPase domain in R1.LlaJI prompted us to investigate whether or not this subunit displayed such an activity. Analysis of GTP hydrolysis, conducted by colorimetric assay (see materials and methods) suggested that R1.LlaJI did not exhibit an intrinsic GTPase activity. Surprisingly, R1.LlaJI dependent GTP hydrolysis could not be activated by the inclusion of "specifc" DNA and/or partially purified R2.LlaJI in the reaction. Similar results were obtained with alternative nucleotides.

### Phenotypic analysis of heterologous LlaJI expression

In an attempt to explain the lack of endonuclease activity in the protein preparations, a phenotypic investigation of the efficacy of LlaJI in the heterologous *E. coli *host was performed. As can be seen from Table 3, no significant resistance to bacteriophage proliferation was observed with a host containing plasmid pJO-J, encoding the complete LlaJI system. A similar phenotype was associated with the host over-expressing R1.LlaJI or R2.LlaJI alone (plasmids pUC-R1 and pUC-R2, respectively).

When both subunits were over-expressed in the same host (plasmid pUC-R1R2) however, and in agreement with results for the lactococcal over-expression host above, a phage resistance phenotype was apparent (Table 3). The degree of this resistance was significantly lower than expected given the number of LlaJI sites on the λ genome and the obvious relationship between EOP and LlaJI site frequency for the lactococcal phage used (see Table 4). The lack of phage resistance associated with the pJO-J containing host was therefore likely attributable to the lower copy number of this replicon, coupled to the lower efficiency of *in vivo *LlaJI restriction in this host.

Investigation of the temperature induced efficacy of LlaJI in *E.coli *further revealed that, although the observed resistance (with plasmid pUC-R1R2 at 30°C) was not evident at 37°C, it was not significantly enhanced with growth at low temperatures (data not shown). Again, as with the lactococcal hosts, crude extracts of *E.coli *over-expressing R1.LlaJI and R2.LlaJI (from plasmid pUC-R1R2) did not exhibit detectable endonuclease activity.

## Discussion

The molecular mechanism by which the lactococcal plasmid-encoded LlaJI R/M system achieves DNA restriction has remained largely elusive. Previous studies have characterized the phage resistance capabilities of this four-gene cluster which is accompanied by an intricate transcriptional regulatory mechanism [1,20].

In the present study we offer additional phenotypic and genetic data which alludes to an unusual mode of action for LlaJI. Analysis of the deduced amino acid sequence for R1.LlaJI revealed a segmented architecture, with a C-terminally located putative GTP binding and hydrolysis domain, and a largely unique N-terminal portion. This organization was similar to that observed for the McrB subunit of the McrBC methyl-specific endonuclease [25,37].

A mutational analysis of the three motifs which comprise the GTPase domain of McrB confirmed the assignment for motif I only, which is the most highly conserved of the three with respect to R1.LlaJI and its homologues (see Fig 1B). Conservative substitutions in motifs II and III failed to unequivocally confirm the proposed assignments on McrB [29]. It was suggested that the proposed motif III, although essential for GTP binding and hydrolysis (N333 in particular), rather than define nucleotide specificity (predicted for D336), was more likely to be involved in McrC interaction (a suggestion corroborated by the significant McrC-stimulated GTPase activity of McrB) [29]. The apparent replacement of the conserved lysine of motif III with a threonine (a divergence also preserved on R1.LlaJI, Fig1A) further substantiated the likelihood of this motif contributing an atypical function.

In addition, the relative spacing between motif's II and III on McrB (29 amino acids), appeared too short to be structurally analogous to the classical GTPases (>49 amino acids)[29,38]. However, in the case of R1.LlaJI, the spacing between these proposed motifs is, although relatively large, more canonical (112 amino acids), and in fact the alignment reveals an alternative candidate for the McrB motif II (although the conserved glycine would be replaced by glutamine, Fig 1B).

It has been shown that it is the N-terminal 160 amino acids of McrB which, like R1.LlaJI did not exhibit significant homology to the database, specified the DNA binding activity of McrBC [37]. This, in addition to the placement of the GTPase domain suggested that an equivalent activity could be attributable to the unique N-terminal portion of R1.LlaJI. We examined these predicted properties for R1.LlaJI and indeed established a specific DNA binding activity for this subunit to LlaJI-site containing DNA fragments.

Interestingly, and in contrast to McrB, R1.LlaJI did not exhibit an intrinsic GTPase activity, which could neither be stimulated by specific DNA nor partially purified R2.LlaJI. This latter finding was again in contrast to McrB, whose intrinsic GTPase activity was stimulated 30-fold by McrC [28]. A putative catalytic centre was identified within the amino acid sequence for R2.LlaJI, which suggested a role for this subunit in substrate strand hydrolysis; however, neither R2.LlaJI nor R1.LlaJI (and combinations of the two) exhibited cleavage or nicking activity.

The absolute requirement for both R1.LlaJI and R2.LlaJI for complete *in vivo *activity was clearly demonstrated (Table 2). A mild phenotype was associated with over-expression of R1.LlaJI alone (in *Lactococcus*), although this was most likely due to DNA binding only, which may have served to partially hinder the phage replication machinery.

Although less dramatic, heterologous expression of R1.LlaJI and R2.LlaJI in *E.coli *provided resistance to phage infection where, again an absolute requirement for both R1.LlaJI and R2.LlaJI was observed (Table 3). The relative poor efficacy of the resistance provided in this host background was attributed to instability of the R2.LlaJI subunit. This subunit, in contrast to R1.LlaJI, could not be observed in over-expressing cell extracts when analyzed by SDS-PAGE.

Purification of R2.LlaJI was only achieved when stabilized by fusion to a carrier protein. It is possible that in this fusion correct folding of the R2.LlaJI subunit had been inhibited, and was not subsequently corrected following the proteolytic removal of the carrier. This could account for our inability to reconstitute an active endonucleolytic complex with R1.LlaJI despite the clearly evident DNA binding activity of the latter.

Furthermore, it is possible that the predicted GTP hydrolyzing activity (which presumably facilitates DNA translocation) of R1.LlaJI would only become apparent following assembly of the "active" heterooligomer on its target DNA. It must be noted however, that an additional lactococcal derived cofactor, proteinacious or otherwise, can not be entirely excluded at this point, despite the absence of endonuclease activity in LlaJI-expressing lactococcal crude extracts.

In summary, LlaJI is a heterooligomeric endonuclease wherein the R1.LlaJI subunit is responsible for determining specificity. It is suggested that R2.LlaJI harbours the catalytic centre for DNA cleavage, whose site, although undetermined, is likely to be defined by the relative "placement" of R2.LlaJI on the target DNA (due to its proposed interaction with R1.LlaJI).

## Conclusion

LlaJI was shown to retain behavioral characteristics of a conventional Type IIA system, in that unmethylated DNA is targeted *in vivo *however; unlike any characterized enzyme of its kind it exhibits striking genetic similarities with the Type IV enzyme McrBC, which is specific for methylated DNA. The presence of an McrBC-like enzyme as a component of an R/M system suggests two mutually exclusive modes of action for these enzyme types whereby they are either in isolation, cleaving methylated DNA (McrBC), or in combination with MTases cleaving unmethylated DNA (LlaJI). Whilst unusual, this is not altogether surprising as such contrasting activities have been described for some conventional endonucleases (DpnI and DpnII) [39,40]. Similarly organized R/M systems were found in the database and together with LlaJI, are likely to represent a new subgroup of the Type II family, combining features of both Type II and Type IV systems. Further biochemical characterization of LlaJI, and indeed its identified and relatively unknown homologues (Fig 1A) will hopefully disclose further revelations regarding this novel and remarkable endonuclease family.

## Methods

### Bacteria, bacteriophage, plasmids, media and growth conditions

Details of bacterial strains, bacteriophage and plasmids used in this study are summarized in Table 1. All *L. lactis *strains were grown in M17 broth (Oxoid ltd., Hampshire, UK) containing 0.5 % glucose at 30°C. *E. coli *was grown at 37°C in Luria-Bertani media [41]. Where appropriate, antibiotics were added as follows: for *L. lactis*, 5 μg ml^-1 ^tetracycline, and 10 μg ml^-1 ^chloramphenicol. For *E. coli*, 100 μg ml^-1 ^ampicilin, 25 μg ml^-1^kanamycin and 10 μg ml^-1 ^chloramphenicol. Induction of the nisin-inducible promoter of pNZ8020 in the NZ9000 host background was achieved by inclusion of nisin (1 ng ml^-1^) in growth media [42]. Induction of Ptac, T5, and Plac promoters of pMal-c2x, pQE60, and pUC19, respectively, was achieved by inclusion of 1 mM IPTG in growth media of their respective *E.coli *hosts. Lactococcal bacteriophage were propagated as described previously [1]. Plaque assays were conducted as described elsewhere [43] and the efficiency of plaquing (EOP) was calculated as the ratio of the number of plaques formed on a tested host to those formed on a sensitive host. Bacteriophage λ.0 was propagated and enumerated using standard techniques [41].

### Molecular techniques

Restriction enzymes and T4 DNA ligase were purchased from New England Biolabs (Hertfordshire, UK), and used according to the manufacturer's instructions. *E.coli *plasmid DNA was isolated using the SV wizard plasmid miniprep kit (Promega, Madison, WI, USA) according to the manufacturer's instructions. Lactococcal plasmid DNA was isolated as described previously [44]. Electroporation of plasmid DNA into *E.coli *was performed using standard techniques [41] and in *L. lactis *as described previously [45]. For routine PCR applications *Taq *DNA polymerase (Qiagen, West Sussex, UK.) was used. For plasmid construction, where high fidelity was required, PCR's were conducted using Phusion DNA polymerase (New England Biolabs) according to the manufacturer's instructions. All PCR products were purified using the Jetquick PCR purification kit (Genomed, Lohne, Germany). The correct orientation and integrity of all constructs was verified by sequencing, performed at MWG Biotech (Ebersberg, Germany). Sequence assembly, verification and analysis were achieved using the seqman program from the DNASTAR package (DNASTAR, Madison, WI.). Database searches were performed using the BLAST suite of programs [46] and the REBASE database [7]. The nucleotide sequence of the LlaJI system is present in the GenBank database; accession no. AY530537.

### Plasmid constructs

For the construction of plasmid pNZ-R1, an *llaJIR1 *encompassing PCR product (coordinates 2999–4782, using primers R1F 5'ggg*ggatcc*catataggaggacaaaataatg'3 and R1R 5'ggg*ctcgag*ccatcctctattcttcagtg'3) was inserted into the BamHI – XhoI sites of pNZ8020. Plasmid pNZ-R2 was constructed by insertion of an *llaJIR2 *encompassing PCR product (coordinates 4769–6031, using primers R2F 5'ggg*ggatcc*gaatagaggatggctctatg'3 and R2R 5'ggg*ctcgag*ctaggaattcgggtattctat'3) into the BamHI – XhoI sites of pNZ8020. Similarly, for plasmid pNZ-R1R2, an *llaJIR1R2 *encompassing PCR product (coordinates 2999–6031, using primers R1F and R2R) was inserted into the BamHI – XhoI sites of pNZ8020.

For plasmid pQE-R1, an *llaJIR1 *encompassing PCR product (coordinates 3017–4772, using primers R1QEF 5'ggg*ccatgg*gaaaaatcgtattacca'3 and R1QER 5'ggg*agatct*ttcttcagtgctaacattatc'3) was inserted into the NcoI – BglII sites of pQE60. For plasmid pQE-R2, an *llaJIR2 *encompassing PCR product (coordinates 4786–6031, using primers R2QEF 5'ggg*ccatgg*aaactaacaaaactattaa'3 and R2QER5'ggg*agatct*ggaattcgggtattctattg) was inserted into the NcoI – BglII sites of pQE60. Plasmid pMal-R2 was constructed by insertion of an *llaJIR2 *encompassing PCR product (coordinates 4786–6031 using primers MalR2F 5'atggaaactaacaaaactattaa'3 and MalR2R 5'ggg*tctaga*ctaggaattcgggtattcta'3) into the XmnI – XbaI sites of pMal-c2x. Plasmid pUC-R1 was constructed by insertion of an *llaJIR1 *encompassing PCR product (coordinates 3003–4775, using primers UCR1F 5'gggg*gtcgac*ttaaggaggacaaaataatgggaa'3 and UCR1R 5'ggg*ggatcc*ctattcttcagtgctaacatta'3) into the SalI – BamHI site of pUC19. For plasmid pUC-R2, an *llaJIR2 *encompassing PCR product (coordinates 4773–6031, using primers UCR2F 5' gggg*gtcgac*ttaagaggatggctctatggaaa'3 and UCR2R 5'ggg*gaatcc*ctaggaattcgggtattctat'3) was inserted into the SalI – BamHI sites of pUC19. Plasmid pUC-R1R1 was constructed by insertion of an *llaJIR1R2 *encompassing PCR product (coordinates 3003–6031, using primers UCR1F and UCR2R) into the SalI – BamHI sites of pUC19.

### Protein expression and purification

R1.LlaJI was expressed as a C-terminal 6xhis tagged derivative, overproduced from plasmid pQE-R1 in the M15/pRep4 host background and purified essentially to homogeneity using Ni-NTA Agarose (Qiagen) affinity chromatography, according to the manufacturer's instructions. R2.LlaJI was overproduced from plasmid pMal-R2 in ER2683 and purified by affinity chromatography using amlyose resin (New England Biolabs), according to the manufacturer's instructions. Purified proteins were subsequently dialyzed against buffer containing 10 mM Tris (pH 7.5), 50 mM KCl, 1 mM DTT, 0.1 mM EDTA and 50 % glycerol, and stored at -20°C. Initial mass and purity estimations were achieved by SDS-PAGE [47] under denaturing conditions. Mass spectrometry and N-terminal amino acid sequencing where indicated was performed at the University of Newcastle Upon Tyne using an Applied Biosystems Voyager DESTR mass spectrometer and a Beckman LF 3000 microsequencer.

### Gel mobility shift analysis

DNA binding assays were carried out in 20 μl volumes containing 50 mM Tris-HCl (pH 7.5), 150 mM NaCl, 5 mM β-mercaptoethanol, 8 % glycerol and 50 μg ml^-1 ^calf thymus DNA and approximately 2.5–5 nM final concentration of a probe. All probes were generated by PCR amplification of a section of the LlaJI promoter (coordinates 309–539 using primers akjF 5'ctggagactatattgctcatcg'3 and P1R 5'gggattgtgtttgaaatcacacc'3) which contained two LlaJI recognition sites. Probe J represented the "wild type" sequence. Disruption of the 5' proximal and 3'proximal sites individually (by C-A replacement of the internal cytosine of 5'GCGTC'3) to produce probes J1 and J2 respectively, was achieved by oligonucleotide-directed mutagenesis. Both sites were disrupted in this manner to produce probe J3, which was devoid of any LlaJI recognition sites. All probes were subsequently end-labelled with (γ-^32^P)ATP by use of polynucleotide kinase (New England Biolabs). Binding reactions were allowed to proceed at room temperature for 30 minutes, followed by the addition of 5 μl 50 % glycerol. Samples were then subjected to electrophoresis on a 4 % polyacrylamide gel containing 2.5 % glycerol in 1 × TAE buffer for 3 hours at 120 V. The protein-DNA complexes were detected by autoradiography of the dried gel.

### DNA Cleavage assays

Crude cell extracts of *L. lactis *strains were prepared from 1 l of culture grown in GM17 broth, concentrated by centrifugation (7000 rpm, 15 min) and washed in lysis buffer (10 mM Tris-HCl (pH 7.6), 1 mM EDTA, 100 mM NaCl and 1 mM DTT). The cell pellets were resuspended in 10 ml of lysis buffer and disrupted with a French press (at 2000 lb/in^2^) after which cell debris and ribosomes were removed by centrifugation (14,000 rpm, 20 min). *E. coli *crude extracts were generated essentially as above from 1 l of culture grown in LB broth, where lysis was achieved by sonication (5 × 10 sec bursts punctuated by 30 sec on ice). Cleavage activity of LlaJI was surveyed in all 4 NEBuffers (New England Biolabs) and at temperatures ranging from 19 – 30°C.

Typically the reactions contained 1 μg substrate DNA (λ DNA and Sk1 DNA for cleavage assays or pBR322 DNA for nicking assays), 100 μg ml^-1 ^BSA, and 2 μl crude extract in addition to 5 mM GTP in a final volume of 20 μl. For assays with purified proteins, typically reactions contained 0.5 μg R1.LlaJI or R2.LlaJI (or combinations of the two at ratios of 5:1, 2:1, 1:1, 1:2, 1:5, R1.LlaJI:R2.LlaJI).

### GTPase assays

GTP hydrolysis activity of R1.LlaJI was investigated via quantification of the release of inorganic phosphate by colorimetric assay based on spectrophotometric quantification of a phosphomolybdate-malachite green complex using the Malachite Green Phosphate Assay kit (Bioassay Systems, Hayward, CA 94545, USA) according to the manufacturer's instructions.

## Competing interests

Financial competing interests: RR, GGW, and DFH are employed at New England Biolabs from which enzymes for routine molecular techniques and certain purification reagents were purchased.

The author(s) declare that there are no competing interests. Non-financial competing interests: The authors declare that there are no non-financial competing interests.

## Authors' contributions

JOD conducted all bioinformatic, genetic, molecular, and biochemical experiments and drafted the manuscript. DFH participated in endonuclease activity screening of crude extracts. GGW, GFF, RR, and DVS participated in data interpretation and critical revision whilst JOD and DVS conceived of the study. All authors have read and approved the final manuscript.

## Figures and Tables

**Table 1:** Bacteria, bacteriophage and plasmids used in this study.

Strains and Plasmids	Revevant characteristic(s)	Reference or source
**Strains**		
*E.coli*		
EC101	Cloning host, Kan^R^.	[48]
ER2683	F'*proA+B+ lacIq*Δ *lacZM15 *mini*Tn10 *(Kan^R^) *fhuA2 *Δ*(lacI-lacA)200glnV44e14-rfbD1? relA1? endA1 spoT1?thi-1 *Δ*(mcrC-mrr)114::IS10*.	NEB collection*
M15	Expression host for pQE 60 derivatives.	Qiagen
*L.Lactis*		
MG1614	Plasmid-free derivative of *L.Lactis *supsp. *lactis *712	[49]
NZ9000	MG1363, *pepN*::*nisRK*, nisin inducible overexpression host.	[42]
NCK690KP1	Partially cured derivative of NCK203. Host strain for phage Ul36	[50]
		
**Bacteriophage**		
λ	*E.coli *temperate bacteriophage.	NEB collection*
Sk1	Small isometric headed phage for MG1614.	[51]
C2	Prolate headed phage for MG1614.	[52]
Ul36	P335 lytic phage for NCK690 KP1.	[53]
		
**Plasmids**		
pPTPi	Low-copy number *E.coli-L.Lactis *shuttle vector for cloning, Tc^R^.	[1]
pJO-J	pPTPi derivative containing complete 6.2 kb LlaJI operon from pNP40.	[1]
pJO-M1M2	pPTPi derivative containing 3.0 kb fragment from pNP40 encompassing promoter, *llaJIM1*and *llaJIM2 *genes.	[1]
pNZ8020	High-copy number *E.coli-L.Lactis *overexpression vector, P*nisA*, Cm^R^.	[54]
pNZ-R1	pNZ8020 derivative containing *llaJIR1 *under control of P*nisA*.	This study
PNZ-R2	pNZ8020 derivative containing *llaJIR2 *under control of P*nisA*.	This study
pNZ-R1R12	pNZ8020 derivative containing *llaJIR1*and *llaJIR2 *under control of P*nisA*.	This study
pUC19	High-copy number *E.coli *cloning vector, Amp^R^.	[55]
pUC-R1	pUC19 derivative containing *llaJIR1*.	This study
pUC-R2	pUC19 derivative containing *llaJIR2*.	This study
pUC-R1R2	pUC19 derivative containing *llaJIR1 *and *llaJIR2*.	This study
pMal-c2x	Medium-copy number expression plasmid	NEB collection*
pMal-R2	pMal-c2x derivative containing *llaJIR2*.	This study
pQE60	High-number 6xhis tag expression plasmid.	Qiagen
pQE-R1	pQE60 derivative containing *llaJIR1*.	This study
pQE-R2	pQE60 derivative containing *llaJIR2*.	This study
pRep4	Low-copy number LacI expressing pQE60 companion	Qiagen
	plasmid.	

* New England Biolabs collection, obtained from M. Sibley and E. Raleigh at NEB.

**Table 2:** LlaJI mediated bacteriophage resistance to the lactococcal phage Sk1.

**Strain/plasmid**	**30°C**		**19°C**	
	**EOP**	**Plaque size after 16 hrs (mm)**	**EOP**	**Plaque size after 16 hrs (mm)**
	
MG1614/pPTPi	1	3	1	2–3
MG1614/pJO-J	3.3 × 10^-3^	3	2.6 × 10^-6^	1–2
NZ9000/pJO-M1M2/pNZ8020	1	3	1	2–3
NZ9000/pJO-M1M2/pNZ-R1	0.7–1	3	0.5–1	1–2 and pinpoint
NZ9000/pJO-M1M2/pNZ-R2	1	1	1	2–3
NZ9000/pJO-M1M2/pNZ-R1R2	1 × 10^-5^	2–3	5 × 10^-6^	1–2 and pinpoint

**Table 3:** LlaJI-mediated resistance to bacteriophage λ. Results reflect values obtained for assays conducted at 30°C.

**Strain/plasmid**	**EOP**
ER2683/pPTPi	1
ER2683/pJO-J	1
ER2683/pJO-M1M2/pUC19	1
ER2683/pJO-M1M2/pUC-R1	1
ER2683/pJO-M1M2/pUC-R2	1
ER2683/pJO-M1M2/pUC-R1R2	7 × 10^-2^

**Table 4:** Relationship between EOP and frequency of LlaJI recognition sites on selected phage genomes.

**Phage**	**EOP**	**Genome size (kb)**	**No. LlaJI sites**
Sk1	3.3 × 10^-3^	28.4	10
C2	1 × 10^-4^	22.1	32
UL36	3 × 10^-2^	36.8	7
Lambda	7 × 10^-2^	48.5	102
